# Clinical trial of a humanized monoclonal anti-IL15Rβ (CD122), in HTLV-1 associated myelopathy/ tropical spastic paraparesis (HAM/TSP)

**DOI:** 10.1186/1742-4690-11-S1-O6

**Published:** 2014-01-07

**Authors:** Raya Massoud, Yoshimi Enose-Akahata, Giovanna S Brunetto, Joan Ohayon, Kaylan Fenton, Irene Cortese, Thomas A Waldmann, Steven Jacobson

**Affiliations:** 1 Viral Immunology Section, Neuroimmunology Branch, National Institutes of Neurological Disorders and Stroke, Bethesda, MD, USA; 2 National Cancer Institute, National Institutes of Health, Bethesda, MD, USA

## 

CD122 is the common beta subunit shared by the receptors for interleukins-2 and -15 (IL-2, IL-15), two cytokines implicated in the immunopathogenesis of HTLV-1 associated myelopathy/ tropical spastic paraparesis (HAM/TSP). Several in vitro findings suggest that CD122 might be a therapeutic target in HAM/TSP: HAM/TSP CD8+ T-cells show increased CD122 expression at baseline and the addition of Humik-ß1, a humanized monoclonal antibody against CD122, to cultures of HAM/TSP peripheral blood mononuclear cells (PBMC) significantly decreases endogenous STAT-5 phosphorylation, spontaneous CD8+T-cell degranulation and spontaneous lymphoproliferation. Based on these findings, we are currently evaluating the safety, clinical and immunological effects of intravenous anti-IL15Rb therapy at 1mg/kg in patients with HAM/TSP. As of today three subjects have been treated at this dose and all showed full saturation of the CD122 receptor. The therapy has been well tolerated and in the single patient who had completed the trial we detected a reduction in the ex vivo CD8 spontaneous degranulation, CD25 and CD56 expression. Notably, the patient also reported resolution of neurogenic bladder symptoms and had objective improvement in Ambulatory Index. Additional treated subjects will be reported to determine the extent of these encouraging preliminary results.

